# Multimodal Bioinspired Self‐Healing Composites Enabling Durable and High‐Efficiency Wearable Perovskite Light‐Emitting Diodes

**DOI:** 10.1002/advs.77039

**Published:** 2026-08-10

**Authors:** Loganathan Veeramuthu, Yu‐Chen Wang, Chun‐Tse Tsai, Yen‐Lin Tseng, Xuan Ting King, Tzu‐Ming Hsu, Zhen‐Li Yan, Fang‐Cheng Liang, Pei‐Yin Chen, Georgy Dosovitskiy, Yehonadav Bekenstein, Hyunjin Lee, Tao Zhou, Chi‐Ching Kuo

**Affiliations:** ^1^ Institute of Organic & Polymeric Materials Department of Molecular Science & Engineering National Taipei University of Technology Taipei Taiwan; ^2^ Department of Materials Science and Engineering National University of Singapore Singapore Singapore; ^3^ Division of Polymer Research Material and Chemical Research Laboratories Industrial Technology Research Institute Hsinchu Taiwan; ^4^ Department of Materials Science and Engineering Technion – Israel Institute of Technology Haifa Israel; ^5^ Department of Biomedical Engineering The Pennsylvania State University University Park Pennsylvania USA

**Keywords:** flexible optoelectronics, perovskite LEDs, quasi‐2D perovskites, self‐healing polymer, wearable electronics

## Abstract

Wearable and flexible optoelectronic devices are rapidly advancing toward human‐interactive applications. However, their operational lifespan is often limited by mechanical damage, interface delamination, and environmental influences. Existing encapsulation methods provide only partial protection and lack autonomous repair capabilities under real‐world conditions. In this study, we present a bioinspired self‐healing polymer (SHP) composite modeled after butterfly wings. This material features a cooperative network of hydrogen bonds, disulfide exchange, and π–π interactions, enabling rapid self‐healing at room temperature without external stimuli. The SHP demonstrates outstanding stretchability (4950%), high toughness (30.47 MJ m^−3^), and environmental resilience, with healing efficiencies of 98% in water, 89% in phosphate buffer saline, and 84% at −5°C. When integrated into emissive layers, SHP‐based light emitting diodes (LEDs) achieve high luminance (9598 cd m^−^^2^) and an external quantum efficiency (EQE) of 10.52%. Additionally, SHP‐encapsulated perovskite‐based integrated SHP LEDs reach a peak EQE of 8.43% and current efficiency of 20.67 cd A^−1^. The flexible, crack‐resistant encapsulation effectively prevents moisture ingress and mechanical failure, maintaining 96% luminance after 400 bending cycles. This self‐sustained ISHP‐based strategy enhances device durability, reduces electronic waste, and supports the development of autonomous, repairable optoelectronic systems for wearable displays, smart textiles, and soft robotics.

## Graphical abstract

Butterfly wing–inspired intrinsically self‐healing polymer interlayers enable flexible perovskite optoelectronics with high luminance, strain‐insensitive stability, and environmental resilience. These hierarchically structured films redistribute mechanical stress, suppress defect formation, and maintain performance under repeated bending, paving the way for next‐generation wearable lighting and display technologies with exceptional durability.

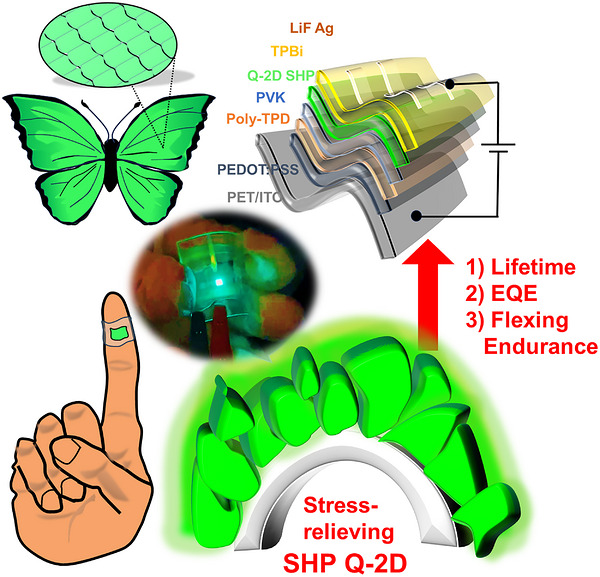

## 1 Introduction

The next generation of wearable and flexible optoelectronic devices demands active materials that can simultaneously deliver high efficiency, mechanical resilience, and long‐term operational stability [1, 2]. Among candidate materials, metal halide perovskites have emerged as front‐runners for light‐emitting diodes (LEDs), photodetectors, and solar cells, owing to their remarkable optoelectronic properties—including high absorption coefficients, long carrier diffusion lengths, narrow emission linewidths, and bandgap tunability across the visible spectrum [3, 4, 5]. Particularly, quasi‐2‐dimensional (Q‐2D) perovskites have gained attention because of their inherent multiple‐quantum‐well (QW) structures, which suppress ion migration and enhance environmental stability compared to their three‐dimensional counterparts [6, 7]. Despite impressive advances in device efficiency, however, flexible and wearable perovskite‐based optoelectronics continue to face formidable barriers to practical application, with mechanical fragility, environmental instability, and interfacial degradation remaining critical bottlenecks.

Flexible devices are especially vulnerable to bending‐induced microcracks, interfacial delamination, and thermal expansion mismatch between functional layers and substrates [8, 9]. These mechanical instabilities are further exacerbated by operational stresses such as Joule heating, exciton–polaron annihilation, and moisture ingress, all of which accelerate trap formation and non‐radiative recombination pathways, thereby reducing device efficiency and shortening lifetimes [10, 11]. The problem is particularly severe in wearable platforms, where repeated mechanical deformation, thermal cycling, and fluctuating environmental exposure are unavoidable. Conventional encapsulation layers provide partial protection against moisture and oxygen, yet they are often rigid, prone to delamination, and incapable of repairing accumulated microdamage [12, 13, 14, 15]. As a result, device luminance, efficiency, and reliability deteriorate rapidly under real‐world operating conditions, highlighting the urgent need for strategies that enable flex‐insensitive performance.

Self‐healing polymers (SHP), inspired by the autonomous repair mechanisms of biological tissues, offer a promising pathway to address these challenges. Unlike extrinsic systems that rely on finite healing agents, intrinsic SHP employ dynamic covalent or non‐covalent interactions—such as hydrogen bonding and disulfide exchange—that can repeatedly restore structural and functional integrity under ambient conditions [16, 17]. In particular, networks combining strong and weak hydrogen bonds with dynamic disulfide linkages create cooperative frameworks capable of balancing mechanical toughness, flexibility, and repeatable healing efficiency [18, 19]. Such systems are inherently sustainable and long‐lived, making them ideal for flexible and wearable electronics [20]. Yet, to date, most SHP applications in perovskite optoelectronics have been limited to encapsulation, with little effort directed toward exploiting their multifunctional potential in device architectures. Integrating autonomous self‐healing chemistry into wearable platforms addresses critical challenges regarding operational durability and fatigue resistance under repetitive physiological deformation, opening promising avenues for stable, long‐term health monitoring and targeted light‐based therapies.

In nature, butterfly wings provide an instructive model for multifunctional resilience. Their cuticle structures display a hierarchical combination of stiff protein‐rich layers for mechanical strength and softer hydrated domains that impart flexibility, toughness, and environmental stability. Inspired by this architecture, we designed a butterfly wing–inspired intrinsically self‐healing polymer that unites the roles of emissive matrix and protective encapsulation within a single multifunctional layer. The SHP integrates three complementary dynamic bonding motifs: strong hydrogen bonds that impart cohesive strength, weak hydrogen bonds that enable chain mobility and reversible flexibility, and dynamic disulfide bonds that restore backbone integrity through covalent exchange at room temperature. This synergistic design yields a polymer with high fracture toughness, rapid ambient‐condition healing (>90% recovery efficiency), and strain tolerance superior to conventional polymer encapsulants.

Our central hypothesis is that embedding Q‐2D perovskite nanostructures within a bioinspired SHP framework enables emissive layers that are simultaneously efficient, mechanically compliant, environmentally robust, and flex‐insensitive. The SHP is designed to (i) regulate perovskite crystallization, promoting preferential orientation and minimizing grain‐boundary defects, (ii) passivate interfacial traps through hydrogen bonding and ligand exchange, and (iii) dissipate mechanical and thermal stresses via reversible bond exchange, thereby preventing irreversible microstructural damage. Unlike conventional device architectures where the emissive medium and encapsulation are separate, our strategy integrates both functions into a single multifunctional layer. This integration simplifies fabrication and enables autonomous in situ repair of microcracks and interfacial defects, providing a robust platform for wearable optoelectronics.

The multifunctionality of the integrated SHP (ISHP) is particularly critical for realizing flex‐insensitive performance, a key benchmark for next‐generation wearable devices. ISHP‐based perovskite LEDs demonstrate ∼96% luminance retention after 400 bending cycles and achieve an operational lifetime (T_50%_) of 310 min—nearly threefold longer than devices encapsulated with conventional SHPs. Beyond electrical durability, the dynamic yet impermeable ISHP network provides strong resistance against moisture ingress, sustaining emission stability under 70% relative humidity. Collectively, these results highlight the ability of ISHP to simultaneously stabilize, protect, and regenerate perovskite emissive layers, effectively bridging the gap between high efficiency and long‐term durability. More broadly, this design introduces a paradigm for unifying emissive and encapsulation functions into a single hybrid layer, advancing sustainable and long‐lived perovskite optoelectronics. We anticipate that this concept can be extended across diverse platforms from flexible displays and skin‐mounted lighting to adaptive optoelectronic textiles paving the way toward practical realization of wearable perovskite technologies.

## 2 Results and Discussions

### 2.1 Design and Synthesis of Mechanically Robust, Self‐Healing Polymers for Wearable Optoelectronics

**FIGURE 1:**
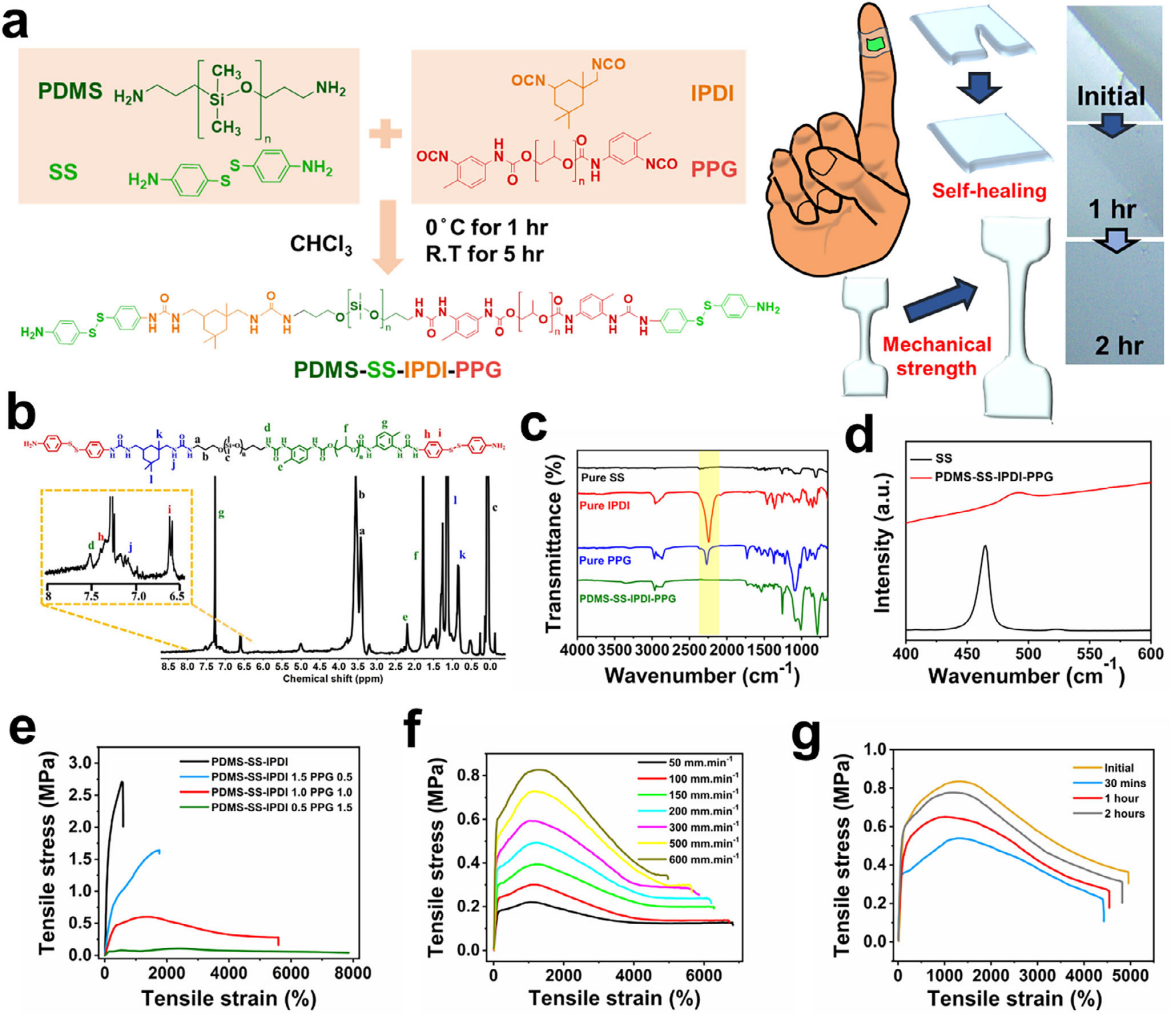
Chemical design, synthesis, and mechanical performance of self‐healing polymers (SHPs) for high‐toughness wearable optoelectronics. (a) Molecular structure and synthetic pathway of the SHP network. (b) NMR plot. (c) FTIR plot. (d) Raman spectra. (e) Tensile strength optimization as a function of monomer ratio. (f) Strain‐rate–dependent stress–strain curves demonstrating mechanical compliance. (g) Rapid room‐temperature self‐healing under high strain rate (600 mm min^−1^).

To develop a self‐healing polymer (SHP) that simultaneously exhibits high mechanical resilience and autonomous repair capabilities, we designed a segmented copolymer system comprising polydimethylsiloxane (PDMS) for enhanced flexibility, disulfide‐containing diamines (SS) for dynamic covalent exchange, isophorone diisocyanate (IPDI) to impart rigidity, and poly(propylene glycol) (PPG) for elasticity. A one‐pot synthesis conducted under ambient conditions produced a structurally dynamic and chemically adaptable SHP with hierarchical bonding domains (Figure 1a). The molecular architecture integrates flexible, rigid, and reversible segments, resulting in high stretchability, toughness, and repeatable healing properties—attributes essential for advanced wearable optoelectronic applications. Proton nuclear magnetic resonance (^1^H NMR) spectroscopy (Figure 1b) validated successful copolymerization: signals corresponding to PDMS methyl protons appeared between 0.1–0.5 ppm, while PPG methylene and methine protons resonated at 3.2–3.8 ppm [21]. Aromatic protons from SS units and hydrogen‐bonded amide (─NH─) groups were observed between 6.5–8.0 ppm, confirming disulfide incorporation and urethane bond formation [22]. The splitting patterns in the aromatic region further supported the integration of SS units. Fourier transformation infrared (FTIR) spectra (Figure 1c) indicated the disappearance of the isocyanate (─NCO) stretch around 2270 cm^−1^ in the final polymer, alongside characteristic urethane N─H (∼3300 cm^−1^) and C═O (∼1700 cm^−1^) absorption bands [23, 24]. The presence of a C─O─C vibration near 1100 cm^−1^ confirmed the incorporation of PPG segments, contributing to the material's flexibility [25]. Raman spectroscopy (Figure 1d) revealed a maintained S─S symmetric stretch near 500 cm^−1^, which was slightly broadened and red‐shifted compared to pristine SS. This shift indicates the integration of disulfide bonds into a more flexible and dynamic chemical environment, enhancing disulfide exchangeability—a critical factor for room‐temperature self‐healing performance.

### 2.2 Dynamic Mechanical Analysis and Strain‐Rate Behavior of SHPs

Tensile testing results demonstrate that molecular design significantly influences the mechanical response (Figure S1). The PDMS–PPG blend exhibited poor mechanical robustness with a tensile strength below 0.02 MPa, whereas incorporation of IPDI significantly improved rigidity to ∼0.07 MPa. A further synergistic enhancement was achieved in the full PDMS–SS–IPDI–PPG formulation, which delivered a tensile strength at break (∼0.10 MPa) and markedly higher elongation due to energy‐dissipative mechanisms arising from reversible disulfide metathesis and dynamic hydrogen bonding. Systematic optimization of PPG content (Figure 1e) identified PDMS–SS–IPDI1.0–PPG1.0 as the optimal composition, exhibiting outstanding mechanical resilience with ∼5590% strain‐at‐break and a tensile strength of 0.28 MPa. Reducing PPG content (0.5) restricted chain mobility and suppressed elongation, whereas excess PPG (1.5) imparted excessive softness and reduced strength. The balanced 1.0:1.0 ratio therefore affords an elastic, tough, and self‐adaptive network that combines flexibility, rigidity, and dynamic reversibility (Table S1). Strain‐rate testing conducted over a range of 50–600 mm min^−1^ (Figure 1f) demonstrated viscoelastic stiffening, with tensile stress increasing from ∼0.2 MPa at slow deformation to about 0.8 MPa at higher rates. This behavior reflects restricted bond reorganization at rapid strain rates, which enhances resistance to breakage, while slower deformation allows bond dissociation and reformation, facilitating energy dissipation. These adaptive mechanical properties are reminiscent of biological tissues and are well‐suited for wearable electronic applications that experience sudden or repetitive deformation. The optimized SHP combines mechanical durability, high stretchability, environmental stability, and intrinsic self‐healing capabilities, supporting the long‐term operation of flexible optoelectronic devices without mechanical failure or performance degradation. Its dynamic bonding network enables rapid recovery from damage, reducing maintenance requirements and extending device lifespan. This bio‐inspired, multifunctional polymer system provides a durable foundation for next‐generation wearable optoelectronic architectures, effectively bridging the gap between structural resilience and adaptive functionality.

### 2.3 Extreme‐Condition Autonomous Self‐Healing Elastomer for Real‐Time Mechanical Resilient Flexible Perovskite Optoelectronics

A primary objective in our SHP development was to engineer an elastomer capable of rapid and efficient recovery of its mechanical and functional properties at ambient temperature without the need for external stimuli, while ensuring robust performance in extreme operational environments. Healing efficiency was evaluated by creating incisions in tensile specimens (Equations (1) and (2)) and monitoring the autonomous recovery over specific time intervals.

(1) $$T=\int_{0}^{\varepsilon }f\lambda \left(\varepsilon \right)d\varepsilon$$

where λ and ε corresponds to the tensile stress and elongation at break. We arrived at the toughness values by utilizing the stress–strain plot of the as‐prepared thin films.

The toughness was calculated by integration of the area under the stress–strain curves. The self‐healing efficiency was calculated as follows:

(2) $$\mathrm{Self}-\mathrm{healing}\;\mathrm{efficiency}\;\%=\frac{{\mathrm{Toughness}}_{\mathrm{healed}}}{{\mathrm{Toughness}}_{\mathrm{fresh}}}\times 100$$

**FIGURE 2:**
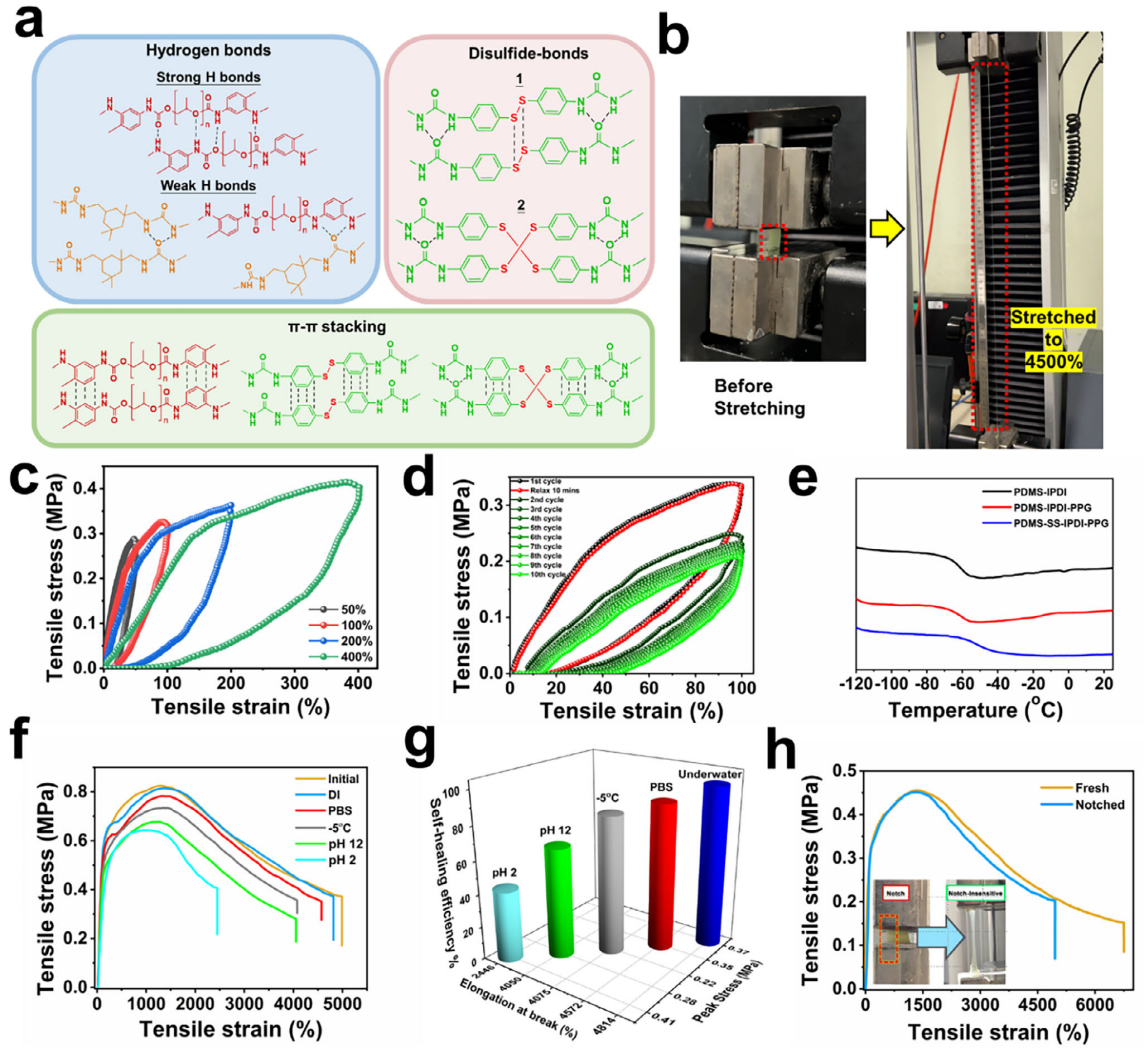
Multimodal self‐healing behavior of SHPs under ambient and extreme conditions. (a) Molecular design enabling simultaneous RT and underwater healing via dynamic bond exchange and hydrophilic domain regulation. (b) Real‐time tensile elongation before fracture and post‐healing recovery. (c) Cyclic tensile profiles at varying elongations. (d) Cyclic stress strain plots. (e) Differential scanning calorimetry thermograms of block‐infused copolymers. (f) Healing efficiency under extreme conditions. (g) 3D self‐repairing‐mapping plots. (h) Notch fracture test demonstrating high fracture toughness (inset: images before and after stretching).

The original polymer demonstrated a maximum tensile strength of 0.83 MPa and a toughness of 30.47 MJ m^−3^. After 30 min of ambient healing, the tensile properties recovered to 59.3%, further increasing to 72.6% after 1 h and reaching 89.5% after 2 h (Figure 1g and Table S2), representing one of the highest reported room‐temperature healing efficiencies for disulfide‐ and hydrogen‐bond‐based SHPs without external activation [26, 27, 28]. This outstanding recovery performance is attributed to a cooperative tri‐modal interaction framework involving strong and weak hydrogen bonds, dynamic disulfide (S─S) bonds, and π–π stacking interactions. The strong hydrogen bonds serve as structural anchors, while weaker hydrogen bonds facilitate chain mobility. Disulfide bonds enable covalent reformation, and π–π stacking provides reversible van der Waals stabilization (Figure 2a). These combined interactions promote localized chain interdiffusion and interfacial reconstruction, enabling crack closure and restoration of structural integrity autonomously—an essential feature for maintaining the performance of flexible perovskite optoelectronic devices under repeated bending and flexural stress [29, 30].

Mechanical testing confirmed the SHP's remarkable toughness and elastic memory. Tensile stress–strain analysis revealed stretchability up to ∼4500% strain (Figure 2b) without failure, a behavior resulting from the synergistic effects of flexible PDMS/PPG segments and reversible SS/IPDI crosslinking domains. The stress–strain curves displayed a gradual increase in stress followed by a broad plateau, indicative of progressive energy dissipation through sequential reversible bond rupture and reformation—similar to the adaptive mechanics observed in biological tissues [31]. Cyclic tensile tests performed at strain levels of 50%, 100%, 200%, and 400% demonstrated stable hysteresis loops (Figure 2c) with minimal softening. Following 10 cycles at 100% strain, the peak stress retention exceeded 95%, indicating rapid chain dynamics and effective relaxation mechanisms (Figure 2d). Differential scanning calorimetry showed a glass transition temperature (T_g_) of −40°C for the PDMS–SS–IPDI–PPG network, markedly lower than that of control systems (Figure 2e), ensuring dynamic bonding activity across a broad thermal window. The flexible PDMS and PPG segments impart temperature‐independent healing and stability, advantageous for outdoor and user‐contact applications. Complementary thermogravimetric analysis confirmed high thermal robustness, with the optimized SHP exhibiting a decomposition temperature (T_95%_) of 282.2°C (Figure S2), highlighting its resistance to thermal degradation while maintaining mechanical adaptability.

Environmental adaptability testing demonstrated the elastomer's capacity for near‐complete recovery under challenging conditions (Table S3). The optimized PDMS–SS–IPDI_1.0_–PPG_1.0_ elastomer achieved underwater healing efficiency of 98%, 89% in phosphate buffer saline solution, and 84% at −5 °C, attributable to hydrophobic PDMS shielding and PPG‐enhanced chain mobility (Figure 2f,g). Even in extreme pH environments (pH 2 and pH 12), the material‐maintained healing efficiencies of 43% and 67%, respectively, despite partial protonation/deprotonation of bonding motifs. Notch propagation assessments indicated a fracture toughness of 14.79 kJ m^−2^ (Figure 2h), exceeding that of typical elastomers and approaching that of natural skin (2.6 kJ m^−2^) [32]. During crack propagation, sacrificial weak bonds dissipated energy, while S─S exchange reactions facilitated network reconstruction behind the crack front, forming a self‐adaptive fracture mitigation mechanism. This combination of environmental resilience, temperature‐independent healing, and high fracture toughness is particularly beneficial for encapsulating perovskite optoelectronic devices, ensuring continued protection against moisture ingress and mechanical degradation under demanding operational conditions.

The SHP exhibits exceptional stretchability, robustness, and autonomous healing capabilities, addressing critical mechanical reliability concerns in flexible quasi‐2D perovskite devices. Its self‐healing polymer can serve as an encapsulant or electrode binder in bio‐inspired architectures with nanostructured surfaces, mitigating localized stress concentrations and preventing delamination, cracks, or electrical and optical failures. Its environmental resilience—including underwater and low‐temperature healing—enhances stability under conditions such as perspiration, humidity, or immersion, supporting longer device lifespans and reducing maintenance. Synthesized simply under ambient conditions, the PDMS–SS–IPDI–PPG polymer features ultra‐high stretchability (∼4500%), fracture toughness (14.79 kJ m^−2^), high toughness (∼30.47 MJ m^−3^), and rapid room‐temperature healing (89.5% in 2 h). Its multi‐modal bonding network comprising hydrogen bonds, disulfide bonds, and π–π interactions—confers elasticity, damage tolerance, and environmental adaptability, making it an ideal material for integrating into butterfly‐inspired flexible perovskite optoelectronic systems to ensure sustained performance amid operational stresses.

### 2.4 Dual‐Functional Self‐Healing Polymers for Phase‐Modulated Quasi‐2D Perovskites

**FIGURE 3:**
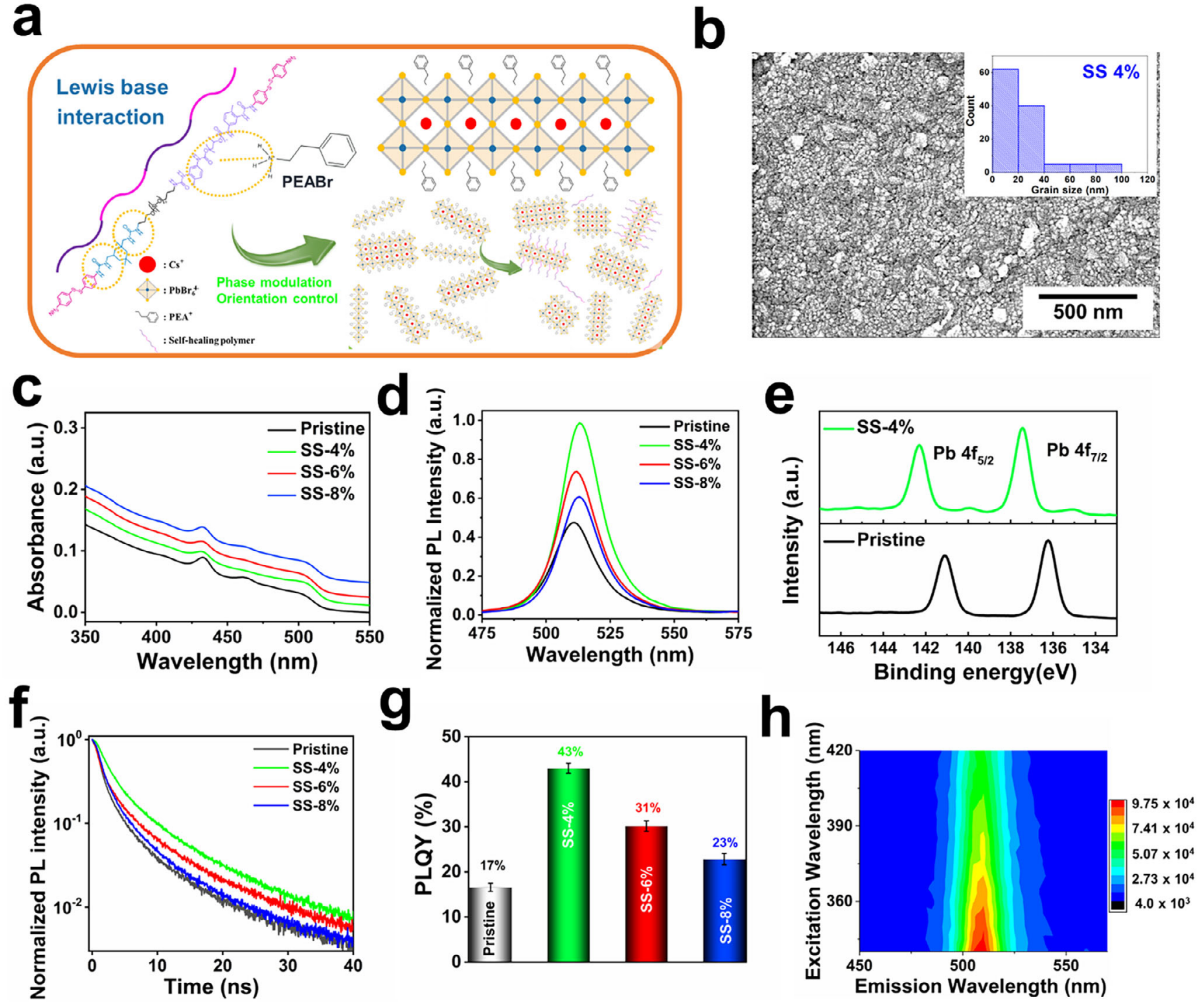
Interfacial modulation and phase control in Q‐2D perovskite–SHP composites. (a) SHP molecular structure and schematic of phase modulation via polymer–perovskite interactions. (b) SEM image with grain‐size distribution inset. (c) UV–vis absorption of SHP‐perovskite. (d) PL spectra of SHP‐perovskite composites. (e) XPS spectra of pristine and SS‐4%. (f) TRPL decay curve. (g) PLQY plot. (h) Excitation–emission mapping of the SS‐4% composite.

Building upon the optimized self‐healing elastomer (SS‐x%) network described in Figures 2 and 3 illustrate the essential function of this elastomeric host material in engineering Q‐2D perovskite thin films with customized structural and optical characteristics. The schematic in Figure 3a depicts the film formation process, wherein the disulfide‐ and urethane‐rich polymer matrix interacts with phenylethylammonium (PEA^+^) cations and PbBr_6_^4−^ octahedra through reversible Lewis base–acid coordination. These dynamic interactions modulate uncontrolled crystallization and guide the vertical stacking of Ruddlesden–Popper (RP) layers, enabling precise control of the n‐value and suppression of defects. Surface morphology analysis (Figure 3b) indicates that the SS‐4% composite forms dense, void‐free grains (29.6 nm), which are smaller and more uniformly distributed compared to pristine (40.4 nm) and higher doped films (SS‐6%: 39.1 nm; SS‐8%: 41.2 nm). Figure S3 shows that surface coverage reaches ∼96% for SS‐4%, surpassing pristine (70.86%) and SS‐8% (72.70%) films. Optical microscopy images illustrating crack closure in the composite film over time (Figure S4) provide direct evidence of morphological self‐healing within the SHP‐perovskite system. This uniform healing indicates the effective dispersion of the SHP matrix within the perovskite, contributing to the performance of the SHP‐perovskite composite without significant deviation from the control SHP. These morphological improvements highlight the dual role of the elastomer as a flexible scaffold and as an interface that suppresses defects, facilitating uniform growth of Q‐2D perovskites—an essential factor for fabricating high‐performance optoelectronic devices.

The optical absorption spectra (Figure 3c) demonstrate a systematic redshift and increased absorption with SS incorporation, peaking at a 4% loading. This suggests improved phase purity and crystallinity facilitated by polymer‐assisted nucleation control, potentially resulting from optimized vertical stacking. Beyond the optimal 4% concentration, excessive polymer content hinders crystal packing due to steric effects and dielectric mismatch, negatively impacting phase uniformity. Steady‐state photoluminescence (PL) spectra (Figure 3d) reflect this trend: SS‐4% exhibits nearly twice the PL emission intensity of the pristine film along with a narrower full width at half maximum (FWHM = 18.3 nm), indicative of reduced non‐radiative recombination pathways and improved perovskite grain uniformity. The PL peak at 513 nm remains consistent across all samples, confirming the absence of low‐dimensional or bulk‐like phases. At higher SS contents, PL intensity diminishes owing to dielectric screening and interfacial scattering caused by the polymer‐rich matrix. Self‐healing capabilities in SS‐4% were demonstrated through in situ PL mapping before and after manual cutting (Figure S5). Physical damage from this process generally causes degradation of the control perovskite films within 2 h. In comparison, the optimized SS‐4% composite film retains 94.2% ± 1.9% of its initial PL emission after two hours at room temperature, while the pristine films show only about 21% recovery under identical conditions. Specifically, the tensile strength at break increased to 0.40 MPa compared to 0.37 MPa for the SHP alone, and the elongation at break decreased slightly to 4738% from 4951%. Figure S6 results confirm that, although the composite is stiffer than the neat SHP likely due to the inorganic lattice—it still maintains its significant healability. Atomic force microscopy (AFM) images (Figure S7) further support these findings, showing that SS‐4% exhibits the lowest surface roughness (Rq = 3.53 nm) and the most tightly packed uniform grains. This nanostructure, characterized by hierarchical ordering reminiscent of bio‐inspired microstructures such as butterfly wings, reduces localized traps and enhances charge transport efficiency.

X‐ray photoelectron spectroscopy (XPS) provides direct evidence of strong interfacial interactions between the self‐healing polymer (SS‐4%) and the perovskite lattice. The Pb 4f_7/2_ and Pb 4f_5/2_ peaks, initially located at 136.22 and 141.12 eV, respectively, exhibit systematic shifts toward higher binding energies (to 137.45 and 142.30 eV) upon SS‐4% incorporation (Figure 3e). These shifts indicate the formation of coordination bonds between Pb^2+^ centers and electron‐donating N atoms of the SHP moieties, which mitigate defect states and suppress nonradiative recombination pathways. Concurrently, Br 3d_5/2_ and Br 3d_3/2_ peaks shift from 66.20 to 67.42 eV and 67.20 to 68.40 eV, respectively, signifying hydrogen‐bond interactions between halide ions and the polymeric network. Such synergistic bonding at both cationic and anionic sites enhances lattice rigidity and inhibits defect migration. Correlating with these electronic signatures, environmental stability assessments under 70% ± 5% relative humidity reveal that SS‐4% stabilized films preserve intense green emission for over four weeks about 82% (Figure S8), in sharp contrast to pristine (35%) or over‐doped counterparts, which rapidly degrade.  This remarkable resilience arises from the hydrophobic, dynamically cross‐linked framework (Figure S9) that retards moisture ingress and prevents ion leaching in quasi‐2D perovskites, while reversible hydrogen bonding, disulfide exchange, and π–π stacking collectively maintain emissive stability under environmental stressors.

Time‐resolved photoluminescence (TRPL) measurements (Figure 3f) reveal an extended average carrier lifetime for the SS‐4% film (τ_avg_ = 6.49 ns) compared to the pristine counterpart (τ_avg_ = 4.35 ns), as calculated from Equations (3) and (4).

Time resolved PL curves were fitted by the biexponential decay function as follows,

(3) $$F\left(t\right)=\sum_{i=1}^{n}A_{i}e^{\frac{1}{t_{i}}}\left(n=2\right)$$

(4) $$\tau_{avg}=\frac{\sum_{i=1}^{n}A_{i}\tau_{i}^{2}}{\sum_{i=1}^{n}A_{i}\tau_{i}}\left(n=2\right)$$

where, *F*(*t*) is the time‐dependent PL intensity; A_1_ and A_2_ are the intensities at *t* = 0; and τ_1_ and τ_2_ are the non‐radiative and radiative components of PL decay lifetime. On solving the Equation (3), we deduce the A_1_. A_2_, τ_1_, and τ_2_ values to calculate the average lifetime using Equation (4).

This prolongation of lifetime indicates efficient trap passivation and enhanced radiative recombination. To substantiate this improvement, photoluminescence quantum yield (PLQY) measurements show a significant increase to 43% ± 1.11% in the SS‐4% film, nearly three times higher than that of the pristine sample (16.6% ± 0.92%) (Figure 3g). Combining the PLQY values and the PL lifetime, the radiative (k_r_) and non‐radiative decay rates (k_nr_) can be calculated according to the Equations (5) and (6).

(5) $$k_{r}=\frac{\mathrm{PLQY}}{\tau_{average}}$$

(6) $$k_{\mathrm{nr}}=\frac{1}{\tau_{average}}-k_{r}$$

Recombination kinetics, extracted from the rate constants k_r_ and k_nr_ (Equations (5) and (6) and Table S4), further confirm an enhanced radiative rate constant (0.07 vs 0.04 ns^−^^1^), highlighting the suppression of nonradiative decay pathways. These combined effects enhance environmental stability and enable precise recombination control, critical for sustained emission performance in perovskite light‐emitting diodes (PeLEDs). Notably, two‐dimensional excitation–emission matrix spectra (Figure 3h and Figure S10) demonstrate spectrally confined emission at 515 nm across a broad excitation window, whereas higher dopant levels induce spectral broadening and increased disorder, compromising color purity. The narrow emission bandwidth evidences effective quantum well confinement and suppression of parasitic low‐dimensional phases, facilitated by SHP‐mediated vertical stacking. Furthermore, the PL stability under continuous excitation attests to the film's resilience under optical stress, aligning with the requirements for flexible, wearable optoelectronic applications. Cytotoxicity assay (Figure S11) of the SHP and SS‐4% against L929 cells demonstrated cell viability rates of over 97% and 88%, respectively, supporting their suitability for wearable applications. Overall, the butterfly‐inspired SHP–perovskite hybrid architecture offers a synergistic platform, integrating controlled morphology, defect passivation, and environmental robustness, advancing the development of high‐performance, self‐healing, flexible PeLEDs.

### 2.5 Structural and Optoelectronic Coupling in Bio‐Inspired SHP–Perovskite Hybrids for High‐Efficiency LEDs

**FIGURE 4:**
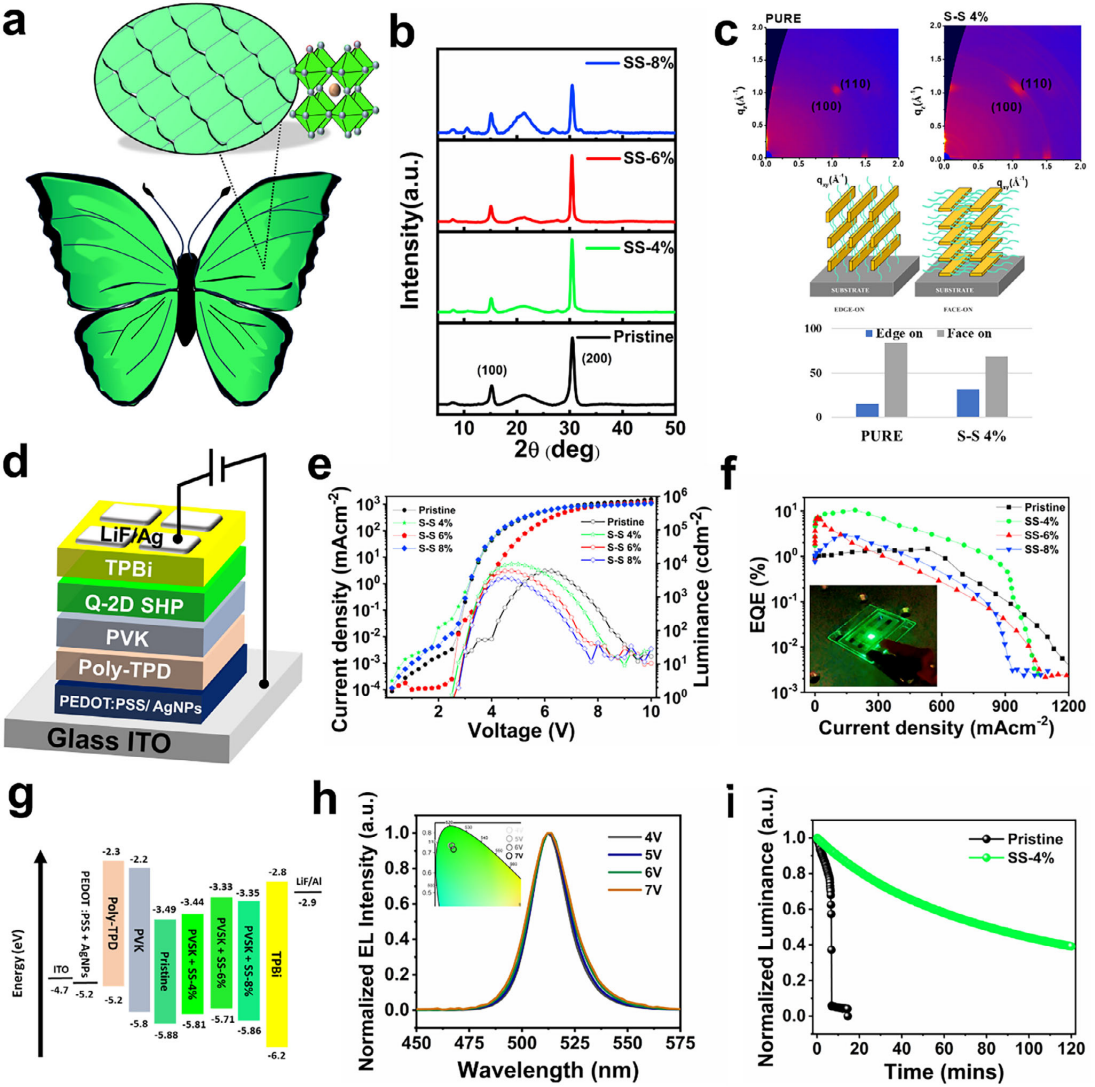
Optoelectronic performance of Q‐2D perovskite–SHP LEDs. (a) Bioinspired emissive layer architecture mimicking butterfly wing photonic structures. (b) XRD profile. (c) GIWAXS profiles confirming crystalline orientation. (d) Rigid LED device architecture schematic. (e) Luminance—current density—voltage profiles of rigid pristine and SHP LED devices. (f) EQE Vs current density of rigid pristine and SHP LED devices. (g) Energy‐level alignment supporting balanced charge injection. (h) EL spectral stability under varying voltages with CIE coordinates (inset). (i) Operational stability showing prolonged luminance retention for SHP LEDs.

The conceptual framework depicted in Figure 4a draws inspiration from the hierarchical microstructures observed in butterfly wing scales, where periodic photonic crystal arrangements enable precise manipulation of optical properties. In our approach, this biological analog is replicated by assembling vertically aligned quasi‐two‐dimensional perovskite nanoplates, organized through π–π interactions between amine and carbonyl functionalities, complemented by directional hydrogen bonding involving phenethylammonium (PEA^+^) spacer cations and the underlying SHP matrix. The polymer‐derived SHP layer, rich in disulfide and urethane groups, plays a dual role: it passivates surface defects and acts as a structural scaffold, promoting lamellar ordering via van der Waals interactions and reversible coordination chemistry. This controlled stacking arrangement results in an anisotropic, layered morphology analogous to natural photonic structures, thereby amplifying light–matter interactions and excitonic confinement—parameters critical for optimized optoelectronic device performance.

X‐ray diffraction (XRD) analysis in Figure 4b confirms the development of well‐defined layered phases within the SHP‐doped perovskite films. The formation of distinct (100) and (200) reflections indicates high crystalline order, with the FWHM of the (200) peak decreasing notably upon 4% SHP doping (SS‐4%), corresponding to an increase in crystallite size from 10.7 to 14.5 nm. Further doping to 6% (SS‐6%) results in an enlarged crystallite size of around 16.4 nm, suggesting improved lattice coherence, likely due to alleviation of interfacial strain. Conversely, higher SHP content at 8% (SS‐8%) reduces the crystallite size to 12.8 nm, indicative of disorder possibly stemming from polymer entanglement or phase segregation. Surface characterization through SEM and AFM (Figure 3b and Figure S7) reveals that SS‐4% films exhibit uniform, smooth grains with minimal voids, whereas increased loading introduces irregularities and roughness. Grazing‐incidence wide‐angle X‐ray scattering (GIWAXS) analysis reveals that the SS‐doped films exhibit a notable enhancement in edge‐on orientation, increasing from 15.5% to 31.6%, as evidenced by the intensified in‐plane (100) and (110) diffraction features (Figure 4c). This strengthened edge‐on alignment promotes more efficient lateral energy and charge transport across the emissive layer. Such improved crystallographic ordering is expected to contribute significantly to the enhanced EQE observed in the bio‐inspired Q‐2D perovskite LEDs.

The incorporation of SHP‐modified perovskite films into PeLED architectures (ITO/PEDOT:PSS/Poly‐TPD/PVK/Q‐2D perovskite:SS x%/TPBi/LiF/Ag) establishes a strong interplay between defect passivation, charge‐transport balance, and mechanical compliance (Figure 4d). Current–voltage–luminance analysis (Figure 4e) reveals that the SS‐4% composition achieves a significantly reduced turn‐on voltage (2.5 V) and a maximum luminance of 9598 cd m^−2^ at 4.75 V, compared with 3.25 V and 6022 cd m^−2^ at 6 V for pristine counterparts. This improvement arises from suppressed non‐radiative recombination, stabilized nanoplatelet orientation, and efficient charge balance. The SHP serves as a dielectric buffer at the perovskite–transport interfaces, alleviating deep trap states while maintaining vertical carrier pathways. In contrast, higher polymer loadings (SS‐6% and SS‐8%) introduce phase segregation and agglomeration, creating charge‐blocking regions that elevate turn‐on voltages and lower luminance (Table S5). External quantum efficiency (EQE) data (Figure 4f) further confirm the optimized polymer content, with SS‐4% devices achieving a peak EQE of 10.52% at ∼176 mA cm^−2^, thirteenfold increase compared to pristine devices (0.81%). Importantly, efficiency roll‐off is mitigated in the SHP‐integrated films, underscoring their ability to sustain radiative recombination under high injection densities. These findings align with PL analyses (Figure 3g–h), which show higher PLQY and narrower emission bandwidth in the SS‐4% blend, linking crystallinity enhancement and orientation control directly to superior device efficiencies.

Electronic structure studies using UPS and optical absorption analysis (Figures S12 and S13 and Table S6) provide further insight into the favorable energy‐level alignment induced by SHP blending. The highest occupied state (HOS) of pristine perovskite (16.44 eV) shifts upward to 16.88 eV in the SS‐4% film, accompanied by a slight narrowing of the optical bandgap from 2.39 eV to 2.37–2.38 eV. This energetic adjustment reduces the interfacial barrier for hole injection while maintaining efficient exciton confinement. In contrast, SS‐8% films display an enlarged bandgap (2.54 eV), reflecting increased structural disorder and reduced orbital overlap at higher polymer content. Optimized SHP integration at 4% thus enables balanced electron and hole injection while suppressing exciton dissociation pathways. Electroluminescence spectra (Figure 4h) further emphasize this effect, with SS‐4% devices exhibiting narrow, stable green emission (CIE x ≈ 0.17, y ≈ 0.74) across 4–7 V, with minimal spectral drift. Pristine and highly doped devices, by contrast, show spectral broadening and blue‐shifted emission, symptomatic of defect‐mediated recombination and phase heterogeneity (Figure S14). The stability and purity of emission in SHP‐integrated films are particularly advantageous for display applications, where color consistency under varying drive conditions is essential for device quality.

Operational testing further validates the role of SHP in improving device reliability. Under sustained electrical bias, the SS‐4% devices maintain nearly 60% of their initial luminance after 120 min, substantially outperforming pristine films, which demonstrate an endurance of only 14 min (Figure 4i). The superior stability derives from the SHP network's multifunctionality: its dynamic bonding dissipates stress and repairs nanoscale defects, while its hydrophobicity and interfacial adhesion suppress ion migration, cation leaching, and delamination—all common failure modes in layered Q‐2D perovskites. These characteristics also correlate with the humidity stability observed in the preliminary result (Figure S8), where SHP‐blended films resist degradation under 70% ± 5% relative humidity. The synergistic combination of defect passivation, energetic alignment, and environmental protection in the optimized SS‐4% architecture establishes a design principle for polymer–perovskite hybrids: balanced polymer content maximizes optoelectronic gains while safeguarding structural integrity. This multifunctional strategy, rooted in a bioinspired self‐healing framework, demonstrates a pathway toward high‐efficiency, color‐stable, and durable flexible LEDs. Beyond efficiency benchmarks, the ability to withstand mechanical deformation and operational stress positions these hybrids as prime candidates for next‐generation wearable photonics and deformable displays, where long‐term stability and emission fidelity are paramount.

### 2.6 Flex‐Insensitive Quasi‐2D Perovskite LEDs Enabled by Integrated Self‐Healing Encapsulation

Flexible perovskite light‐emitting diodes require encapsulation schemes that not only protect against moisture and oxygen but also provide mechanical compliance and preserve optoelectronic properties under continuous mechanical stress. Conventional polyethylene (PE) films, widely adopted due to their simple lamination and low processing cost, offer moderate barrier protection and mechanical adaptability [33]. However, their inherent drawbacks limit long‐term reliability: PE exhibits a relatively high water vapor transmission rate compared to inorganic/organic multilayer barriers, weak interfacial adhesion with perovskite and transport layers, and a lack of recovery capability once microcracks form. These microcracks, generated under cyclic bending, not only serve as initiation sites for delamination but also enable pathways for ion migration, exacerbating nonradiative recombination and accelerating luminance decay [34, 35]. Furthermore, the high‐temperature lamination required for PE sealing is incompatible with flexible substrates and often introduces strain‐induced disorder in Q‐2D perovskite emitters. Given the extreme sensitivity of Q‐2D structures to interfacial disruptions, such encapsulation‐induced stress becomes a major degradation pathway. These shortcomings highlight the need for a multifunctional encapsulation strategy capable of simultaneously maintaining mechanical resilience, suppressing environmental ingress, and safeguarding the electronic landscape of the emissive layer—all while being compatible with scalable, low‐temperature processing. WVTR measurements were conducted on encapsulation films of approximately 80 µm thickness, yielding values of 0.61 g m^−2^ day^−1^ for SHP and 0.85 g m^−2^ day^−1^ for PE. The enhanced moisture barrier of the ISHP encapsulation is due to its densely crosslinked PDMS‐rich network and reversible disulfide/urethane interactions, which reduce free volume and effectively inhibit water vapor diffusion. This improved barrier performance aligns with greater device stability. Such high WVTR values are expected to be advantageous for perovskite flexible LED fabrication.

**FIGURE 5:**
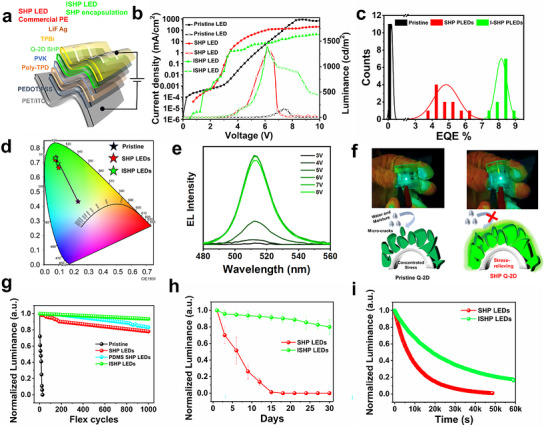
Device performance and stability of flex‐insensitive perovskite LEDs under mechanical and operational stresses. (a) Schematic device architectures of pristine, SHP, and ISHP LEDs. (b) Luminance—current density—voltage profiles of flexible LED devices. (c) EQE Vs current density of flexible LED devices. of the corresponding devices. (d) CIE coordinate stability. (e) Electroluminescence (EL) spectra of ISHP LEDs under bias. (f) Real‐time EL images demonstrating uniform emission under mechanical deformation. (g) Luminance retention during 1000 bending cycles. (h) Ambient stability (70% RH, 25°C) over 30 days. (i) Operational stability of ISHP LEDs under constant electrical driving (Initial luminance of 200 cd/m^2^).

To overcome these limitations, we developed an integrated self‐healing polymer (ISHP) encapsulation strategy, replacing PE with a crosslinked polymer network cured at 120°C within 4 min. The ISHP acts as both a structural barrier and a functional stress‐dissipating layer. Its dynamic hydrogen‐bonding motifs and π–π stacking domains facilitate nanoscale structural recovery following deformation, preventing irreversible crack formation. Comparative device architectures were examined to elucidate the role of self‐healing polymers in flexible Q‐2D PeLEDs: (i) pristine devices encapsulated with conventional PE, (ii) SHP‐LEDs in which the polymer is incorporated solely as an emissive layer additive, and (iii) ISHP‐LEDs where the polymer functions dually as emissive modifier and structural encapsulant (Figure 5a). The complete fabrication workflow, highlighting the distinctions between SHP and ISHP integration strategies, is provided in Figure S15. Current density–voltage–luminance characteristics (Figure 5b and Table S7) show that ISHP devices exhibit low turn‐on voltage (∼2.75 V) and suppressed leakage current relative to SHP‐only devices. ISHP‐LEDs deliver a peak luminance of 1410 cd m^−2^ at 6.25 V, with current efficiency (20.67 cd A^−1^) and external quantum efficiency (8.43%) maintained at maximum brightness. By comparison, SHP‐only devices achieve ∼1310 cd m^−2^ but with significantly reduced efficiency (CE = 1.17 cd A^−1^, EQE = 6.10%), underscoring the role of ISHP encapsulation in minimizing non‐radiative recombination and improving charge balance. The EQE histogram (Figure 5c) confirms a 27‐fold enhancement for ISHP devices over PE‐sealed controls, reflecting the combined benefits of emissive‐layer passivation and polymer‐assisted mechanical integration. EL emission spectral analysis (Figure 5d) shows that ISHP maintains stable saturated green emission (CIE coordinates 0.079, 0.729), with minimal drift (<Δx = 0.011, Δy = 0.022) up to 8 V bias. EL spectra (Figure 5e and Figure S16) remain spectrally narrow (FWHM ∼19.6–20.5 nm) across applied voltages, whereas PE‐encapsulated controls display spectral broadening and instability, indicative of bias‐induced morphological reorganization. These results demonstrate that ISHP encapsulation suppresses phase segregation and preserves emissive‐domain orientation, leading to superior spectral stability.

Mechanical, environmental, and operational testing further highlight the multifunctionality of ISHP encapsulation. Under cyclic bending (bending radius = 5 mm, frequency = 1 Hz), ISHP‐LEDs retain 93.43% of initial luminance after 1000 cycles, compared to 77.97% in SHP LEDs (Figure 5f–g). To deconvolute the contributions of mechanical encapsulation from intrinsic self‐healing, a non‐healable PDMS control encapsulant was tested. The incremental improvement from 83.20% (non‐healing encapsulant) to 93.43% (ISHP) is attributed specifically to the autonomous crack‐repair capability of the dynamic disulfide and hydrogen‐bond network. This clearly demonstrates that mechanical compliance alone accounts for ∼5.2% improvement, while the dynamic self‐healing chemistry of ISHP accounts for the additional ∼10.2% improvement. The EQE decreases marginally from 8.40% to 7.96%, while current efficiency drops only slightly from 20.67 to 20.02 cd A^−1^, underscoring the SHP's ability to homogenize strain distribution and prevent nanoscale defect growth. FE‐SEM images (Figure S17) of the flex‐endured samples were captured, and the results clearly illustrate numerous interconnected microcracks and extensive crack propagation throughout the emissive layer. In contrast, the SHP‐containing devices exhibit a predominantly continuous morphology with significantly fewer defects.

Environmental stability is also enhanced: after 30 days at 70% ± 5% relative humidity, ISHP‐LEDs maintain 79.93% of their initial luminance, whereas SHP devices retain ∼26% within a 9‐day period (Figure 5h). This improvement arises from the hydrophobic crosslinked backbone and well‐organized polymer morphology, which act as robust barriers to moisture and oxygen diffusion. Operational stability measurements (Initial set luminance of 200 cd/m^2^) further confirm the advantage of ISHP integration, with ISHP‐LEDs achieving a T_50%_ lifetime of ∼18 600 s (∼310 min) under constant current operation, over four times longer than SHP‐only devices (∼74 min) (Figure 5i). The extended lifetime is attributed to the polymer's dual function of dissipating electrical/thermal stress and preserving emissive‐layer morphology, while simultaneously maintaining charge‐injection balance. To ensure the safety regarding lead leaching, Inductively Coupled Plasma Optical Emission Spectroscopy (ICP‐OES) analysis was conducted. The measured lead (Pb) concentration was 1.83 µg/L, which is below the World Health Organization (WHO) guideline value of 10 µg/L for drinking water. For comparison, a pristine device subjected to the same test exhibited a lead concentration of 145 µg/L. These results confirm that the SHP significantly reduces lead leakage, even following mechanical damage (Figure S18). Table S8 benchmarks recent advances in flexible perovskite LEDs, highlighting the superior performance metrics achieved through the ISHP strategy. Collectively, these results establish ISHP encapsulation as a comprehensive solution to the long‐standing challenges of flexible perovskite LEDs: combining defect passivation, environmental shielding, and mechanical resilience in a single processable platform. By uniting emissive‐layer stabilization with encapsulation functionality, ISHP provides a pathway toward next‐generation flexible and wearable PeLEDs that demand both efficiency and durability under operational stress.

## 3 Conclusion

In summary, ISHP‐integrated Q‐2D PeLEDs demonstrate high efficiency (EQE = 8.43%), robust mechanical durability (96% luminance retention after 400 bending cycles), strong environmental resilience (∼80% luminance retention after 30 days at 70% relative humidity), and extended operational stability (T_50%_ of ∼310 min). These performance metrics surpass those of both SHP‐modified and PE‐encapsulated control devices. The multifunctional encapsulation approach—integrating self‐healing chemistry, phase‐controlled emissive alignment, and hydrophobic barrier properties effectively addresses the mechanical, environmental, and electronic degradation pathways that have typically limited the commercial viability of flexible perovskite LEDs. The room‐temperature curing process enhances compatibility with a variety of flexible substrates, minimizing thermal stress and defect formation associated with traditional PE lamination techniques. Our findings demonstrate that ISHP encapsulation provides promising and potentially scalable solution for producing flexible, environmentally stable, and high‐performance perovskite LEDs, with promising applications in next‐generation wearable displays, foldable devices, and deformable photonic systems.

## 4 Experimental Section

### 4.1 Materials

All commercial materials were purchased and used without further purification. Chemicals and corresponding details are provided in parentheses: Poly(dimethylsiloxane), bis(3‐aminopropyl) terminated (NH_2_–PDMS–NH_2_) (Mn ∼2500 g/mol, Sigma–Aldrich); Bis(4‐aminophenyl) disulfide (SS) (Mn ∼248.37 g/mol, Sigma–Aldrich); Isophorone diisocyanate (IPDI) (Mn ∼222.28 g/mol, 98%, Sigma–Aldrich); Poly(propylene glycol), tolylene 2,4‐diisocyanate terminated (PPG) (Mn ∼2300 g/mol, Sigma–Aldrich); Chloroform (anhydrous, 99.8%, Sigma–Aldrich); Lead(II) bromide (PbBr_2_, ≥98%, Sigma–Aldrich); Phenylethylammonium bromide (PEABr, ≥98%, Sigma–Aldrich); Cesium bromide (CsBr, 99.99%, Sigma–Aldrich); Dimethylformamide (DMF, ≥99.8%, Sigma–Aldrich); Dimethyl sulfoxide (DMSO, ≥99.9%, Sigma–Aldrich); Cleaning agents (isopropanol, ethanol, acetone, Sigma–Aldrich); Indium tin oxide (ITO)–coated glass substrates (Luminescence Technology Corp.); PEDOT:PSS (Clevios P Al 4083); Poly(N,N′‐bis(4‐butylphenyl)‐N,N′‐bis(phenyl)benzidine) (Poly‐TPD, UniRegion Bio‐tech); Poly(9‐vinylcarbazole) (PVK, UniRegion Bio‐tech); 1,3,5‐tris(1‐phenyl‐1H‐benzimidazol‐2‐yl)benzene (TPBi, UniRegion Bio‐tech); Lithium fluoride (LiF, Sigma–Aldrich); Aluminum pellets (Al, Summit‐Tech Co.); polyethylene terephthalate (PET and PET/ITO, Well being enterprise Co. ltd.), polyethylene (PE, Industrial Technology Research Institute, Taiwan).

### 4.2 Preparation of Quasi‐2D Perovskite–SHP Blend Precursor

Quasi‐2D perovskite precursors were prepared by dissolving CsBr, PbBr_2_, and PEABr in a molar ratio of 1.2:1:0.5 in DMSO (0.2 m) and stirred for 3 h. Separately, the SHP solution was dissolved in DMF at different weight ratios (4%, 6%, and 8%). The SHP solution was subsequently blended with the pre‐stirred perovskite precursor and allowed to react overnight at 50°C under continuous stirring, yielding homogeneous quasi‐2D perovskite–SHP blend precursors suitable for device fabrication.

### 4.3 SHP Q‐2D LED Rigid Device Fabrication

ITO‐coated glass substrates were sequentially cleaned by ultrasonication in deionized water, followed by UV–ozone treatment for 10 min. A PEDOT:PSS–Ag nanoparticle mixture (0.45 mm) was spin‐coated (4500 rpm, 45 s) and annealed at 130°C for 15 min. Subsequently, Poly‐TPD (7 mg mL^−1^, chlorobenzene) and PVK (6 mg mL^−1^, chlorobenzene) were deposited sequentially (4500 rpm, 45 s) and annealed at 130°C and 150°C, respectively. The perovskite precursor was spin‐coated (3000 rpm, 60 s, annealed at 80°C, 15 min). Finally, TPBi (30 nm), LiF (1 nm), and Al (100 nm) layers were deposited by thermal evaporation (∼1 × 10^−5^ Pa). Devices (active area: 2 mm^2^) were encapsulated with PE/glass and completed in a N_2_ glove box.

### 4.4 Flexible SHP LED Device Fabrication

Flexible devices were fabricated following identical protocols used for rigid SHP Q‐2D LEDs, except that commercial PET/ITO substrates (98% transmittance, 15 Ω sq^−1^ sheet resistance) were employed as flexible electrodes. Devices were encapsulated using thermoplastic PE sealants with PET encapsulation sheets. The encapsulation was performed at 120°C for 4 min to ensure robust adhesion and protect the device integrity during bending and repeated flexions. This flexible encapsulation strategy enabled reliable device performance under mechanical deformation.

### 4.5 Integrated SHP (ISHP) LED Device Fabrication

Integrated SHP–LEDs were fabricated following the same protocols as rigid and flexible control devices, with the exception of the encapsulation step. Instead of conventional PE sealant, a SHP sealant was employed. The SHP encapsulant was cured at 60°C for 4 min and subsequently laminated with a PET support layer. The lamination was performed at a moderate temperature, avoiding the need for high‐temperature thermal annealing. This process yielded a soft encapsulation layer that ensured mechanical durability, preserved flexion‐insensitive operation, and enhanced device integrity, thereby providing a reliable platform for high‐performance wearable optoelectronic applications.

### 4.6 Characterization

The morphological features of the quasi–two‐dimensional (Q‐2D) perovskite and SHP‐blended films were examined using field‐emission scanning electron microscopy (SEM, JEOL JSM‐6700F), while nanoscale surface topography was further analyzed by atomic force microscopy (AFM, Bruker Innova). Grazing‐incidence wide‐angle X‐ray scattering (GIWAXS) measurements were carried out at beamline BL23A1 of the National Synchrotron Radiation Research Center (NSRRC), Taiwan, to probe crystallographic orientation and phase distribution, complemented by X‐ray diffraction (XRD) for bulk structural evaluation. Proton nuclear magnetic resonance (^1^H NMR, Bruker Fourier 300 MHz) with TMS as the internal standard and Fourier‐transform infrared spectroscopy (FTIR, Bio‐Rad 155) were employed to investigate molecular structure, functional groups, and polymer–perovskite interactions. Ultraviolet photoelectron spectroscopy (UPS, PerkinElmer PHI 5400, Specs UVS 10/35) was used to evaluate energy‐level alignment. The optical properties of pristine and SHP‐blended perovskite films were systematically investigated using UV–vis absorption spectroscopy (Shimadzu UV‐3150), steady‐state photoluminescence (PL, HORIBA FluoroMax‐2 with Xe lamp excitation), photoluminescence excitation (PLE, Horiba Nanolog), and photoluminescence quantum yield (PLQY, Hamamatsu C9920‐01 integrating sphere). Time‐resolved photoluminescence (TR‐PL) decay dynamics were recorded using a Hamamatsu C10910 streak camera with an M10913 slow single‐sweep unit at NSRRC. Raman spectroscopy (JASCO 5100, λ = 532 nm) was performed with Si calibration (520 cm^−^^1^) to probe vibrational modes and lattice interactions. The electroluminescence (EL) properties of Q‐2D LEDs were evaluated using a calibrated PR system comprising a Keithley 2400 source meter, PR670 spectroradiometer, and Minolta CS200 luminance meter, enabling precise current–voltage–luminance characterization. Contact angle and surface wettability were determined using a goniometer (Sindatek 100SB). Mechanical robustness and viscoelastic behavior of the SHP–perovskite composites were analyzed by dynamic mechanical analysis (DMA, EXSTAR 6000, Techmax), while tensile testing was conducted using a QC 508 universal testing machine (Cometech, Taiwan). In addition, a customized in‐house mechanical testing setup was employed to monitor device integrity under repeated bending cycles. Cell viability of the SHP samples was measured via UV–vis absorbance using the MTT assay for durations of 24 and 48 h. The MTT reagent was added in the dark and incubated in a cell incubator. Following incubation, the supernatant was extracted, and the liquid from the culture dishes was monitored using a UV–vis spectrophotometer. SHP emissive layers were scratched with a standardized blade and subsequently immersed in deionized water for 24 h. Inductively coupled plasma optical emission spectroscopy (ICP‐OES) analysis of the resulting water was performed.

## Author Contributions

**Loganathan Veeramuthu**: methodology, investigation, software, data curation, formal analysis, Writing – review and editing, Writing – original draft. **Fang‐Cheng Liang**: data curation. **Zhen‐Li Yan**: formal analysis, data curation, methodology. **Tzu‐Ming Hsu**: formal analysis, data curation, methodology. **Chi‐Ching Kuo**: methodology, investigation, writing – original draft, writing – review and editing, software, formal analysis, data curation, supervision. **Chun‐Tse Tsai**: methodology, formal analysis, data curation. **Xuan Ting King**: formal analysis, data curation. **Tao Zhou**: data curation, formal analysis, writing – review and editing. **Yu‐Chen Wang**: methodology, data curation, software, formal analysis. **Georgy Dosovitskiy**: formal analysis, data curation. **Hyunjin Lee**: formal analysis, data curation. **Pei‐Yin Chen**: data curation, formal analysis. **Yen‐Lin Tseng**: methodology, formal analysis, data curation. **Yehonadav Bekenstein**: data curation, formal analysis, writing – review and editing, methodology.

## Conflicts of Interest

The authors declare no conflicts of interest.

## Supporting information




**Supporting File**: advs77039‐sup‐0001‐SuppMat.docx.

## Data Availability

The data that support the findings of this study are available from the corresponding author upon reasonable request.
