# Supplementary material for: Multimodal Bioinspired Self‐Healing Composites Enabling Durable and High‐Efficiency Wearable Perovskite Light‐Emitting Diodes
Source: Adv Sci (Weinh). 2026 Aug 10:e77039. Online ahead of print. doi: 10.1002/advs.77039 (PMC13455835; doi:10.1002/advs.77039)
Supplement: Supplementary file 1 [file ADVS-9999-e77039-s001.docx]

***Supporting information***

**Multimodal Bioinspired Self-healing Composites Enabling Durable and High-Efficiency Wearable Perovskite Light-Emitting Diodes**

Loganathan Veeramuthu^a^, Yu-Chen Wang^a^, Chun-Tse Tsai*^a^*, Yen-Lin Tseng*^a^*, Xuan Ting King*^a^*, Tzu-Ming Hsu***^a^***, Zhen-Li Yan***^a^***, Fang-Cheng Liang^a,b^, Pei-Yin Chen^c^, Georgy Dosovitskiy^d^, Yehonadav Bekenstein^d*^, Hyunjin Lee^e^, Tao Zhou^e*^, Chi-Ching Kuo^a*^

*^a^Institute of Organic & Polymeric Materials, Department of Molecular Science & Engineering, National Taipei University of Technology, Taipei 10608, Taiwan.*

*^b^Department of Materials Science and Engineering, National University of Singapore, 9 Engineering Drive 1, Singapore 117575, Singapore.*

*^c^Division of Polymer Research, Material and Chemical Research Laboratories, Industrial Technology Research Institute, Taiwan.*

*^d^Department of Materials Science and Engineering, Technion - Israel Institute of Technology, Haifa 3200003, Israel.*

*^e^Department of Biomedical Engineering, The Pennsylvania State University, PA, 16802, USA*

^*^Author to whom all correspondence should be addressed

Tel: 886-2-27712171*2407	Fax: 886-2-27317174

Correspondence to:

Prof. C.-C. Kuo (E-mail: [kuocc@mail.ntut.edu.tw](mailto:kuocc@mail.ntut.edu.tw))

Prof. T. Zhou (E-mail: [tzz5199@psu.edu](mailto:tzz5199@psu.edu))

Prof. Y. Bekenstein (E-mail: [bekenstein@technion.ac.il](mailto:bekenstein@technion.ac.il))

**Supporting Information Figures**

**Fig. S1.** Stress-strain curve of self-healing polymer constituent monomers.

**Fig. S2.** Thermogravimetric analysis curve of PDMS-SS-IPDI-PPG.

**Fig. S3.** (a-d) SEM images of pristine and other SHP composite (SS-4%, SS-6%, SS-8%) and

(e-h) corresponding grain size distribution plots.

**Fig. S4.** Self-healing ability of SS-4% composite under optical microscopic observation.

**Fig. S5.** In-situ PL mapping before and after healing after 2 hours.

**Fig. S6.** Stress-Strain plot of SHP Vs SS-4% composite.

NOTE: for the sake of comparison between SHP and SHP perovskite composites, we have utilized the control SHP of thickness 250 μm and we have spin coated with the optimized SS-4% emissive layer composite over the SHP substrate.

**Fig. S7.** AFM images of (a) pristine (b) SS-4% (c) SS-4%, and (d) SS-8%.

**Fig. S8.** Photoluminescent emission stability over 1 month @ 70 % RH and RT (a) real time UV-exposed thin films and PL emission retention.

**Fig. S9.** Contact angle image of (a) pristine and (b) SS-4%.

**Fig. S10.** 2-D excitation–emission matrix maps of (a) pristine (b) SS-4% (c) SS-4%, and (d) SS-8%.

**Fig. S11.** Cell viability of SHP and SS-4% thin films.

**Fig. S12.** UPS plots of (a) pristine (b) SS-4% (c) SS-4%, and (d) SS-8%.

**Fig. S13.** Tauc plots of (a) pristine (b) SS-4% (c) SS-4%, and (d) SS-8%.

**Fig. S14.** EL spectra and CIE color coordinate diagram of (a,e) pristine (b,f) SS-4% (c,g) SS-4%, and (d,h) SS-8%.

**Fig. S15.** Flexible LED device fabrication steps for SHP LED and ISHP LEDs.

**Fig. S16.** EL spectral operational voltage stability of (a) Pristine LED (b) SHP LED and (c) ISHP LED.

**Fig. S17.** FE-SEM morphology of emissive layers from the flexible devices (a) Pristine and (b) SS-4% sample.

**Fig. S18.** ICP-OES lead leakage test from the flexible devices upon fracture and immersion.

**Table S1.** Self-healing monomer ratio optimization and its respective mechanical properties.

| **Samples** | **Stress at break (MPa)** | **Elongation at break (%)** | **Toughness (MJ m^-3^)** |
| --- | --- | --- | --- |
| PDMS-SS-IPDI | 2.70 | 578.03 | 12.08 |
| PDMS-SS-IPDI_1.5_ PPG_0.5_ | 1.64 | 1745.91 | 20.44 |
| PDMS-SS-IPDI_1.0_ PPG_1.0_ | 0.28 | 5590.90 | 23.40 |
| PDMS-SS-IPDI_0.5_ PPG_1.5_ | 0.04 | 7854.97 | 5.60 |

**Table S2.** Self-healing polymer’s mechanical properties and its room temperature healing characteristics.

| **Samples** | **Stress at break (MPa)** | **Elongation at break (%)** | **Toughness**  **(MJ m^-3^)** | **Self-healing efficiency (%)** |
| --- | --- | --- | --- | --- |
| Initial | 0.36 | 4950.69 | 30.47 | - |
| RT- 30 mins | 0.22 | 4404.44 | 18.06 | 59.27 |
| RT- 1hour | 0.26 | 4542.31 | 22.12 | 72.59 |
| RT- 2 hour | 0.31 | 4815.22 | 27.26 | 89.46 |

**Table S3.** Self-healing polymer’s mechanical properties and its extreme condition healing characteristics.

| **Samples** | **Stress at break (MPa)** | **Elongation at break (%)** | **Toughness**  **(MJ m^-3^)** | **Self-healing efficiency (%)** |
| --- | --- | --- | --- | --- |
| DI | 0.37 | 4814 | 29.82 | 97.67 |
| PBS | 0.35 | 4572 | 27.17 | 88.99 |
| -5^o^C | 0.22 | 4075 | 25.23 | 83.62 |
| pH 12 | 0.28 | 4050 | 20.34 | 66.62 |
| pH 2 | 0.41 | 2446 | 13.17 | 43.14 |

**Table S4.** Detailed decay parameters of TRPL lifetime measurements.

| **Samples** | **A1** | **τ_1_**  **_(ns)_** | **A2** | **τ_2_**  **_(ns)_** | **τ _average_**  **_(ns)_** | **PLQY** | **k_r_**  **_(ns_^-1^_)_** | **k_nr_**  **_(ns_^-1^_)_** | **K_nr_/k_r_** |
| --- | --- | --- | --- | --- | --- | --- | --- | --- | --- |
| Pristine | 0.93 | 1.14 | 0.13 | 7.74 | 4.35 | 16.57 | 0.04 | 0.19 | 5.03 |
| SS-4% | 0.90 | 2.45 | 0.19 | 10.82 | 6.49 | 42.97 | 0.07 | 0.09 | 1.32 |
| SS-6% | 0.80 | 1.30 | 0.25 | 7.04 | 4.91 | 30.15 | 0.06 | 0.14 | 2.32 |
| SS-8% | 0.95 | 1.62 | 0.14 | 8.28 | 4.48 | 22.82 | 0.05 | 0.17 | 3.38 |

**Table S5.** Q-2D based rigid LED device performance.

| **Samples** | **Turn on Voltage (V)** | **Max EL Peak (nm)** | **Max Luminance (cd m^-2^)** | **Current efficiency (cd A^-1^)** | **Max EQE (%)** | **CIE x** | **CIE y** |
| --- | --- | --- | --- | --- | --- | --- | --- |
| Pristine | 3.25 | 514 | 6022 | 2.49 | 0.81 | 0.084 | 0.734 |
| SS-4% | 2.50 | 512 | 9598 | 5.45 | 10.52 | 0.080 | 0.733 |
| SS-6% | 3.00 | 514 | 6024 | 4.52 | 1.48 | 0.082 | 0.736 |
| SS-8% | 3.00 | 514 | 3689 | 2.60 | 0.83 | 0.090 | 0.739 |

**Table S6.** UPS and Tauc plot derived HOMO-LUMO and band gag (Eg) values.

| **Samples** | **HOMO (eV)** | **Eg (eV)** | **LUMO (eV)** |
| --- | --- | --- | --- |
| Pristine | -5.88 | 2.39 | -3.49 |
| SS-4% | -5.81 | 2.37 | -3.44 |
| SS-6% | -5.71 | 2.38 | -3.33 |
| SS-8% | -5.78 | 2.54 | -3.24 |

**Table S7.** Q-2D based flexible LED device performance.

| **Samples** | **Turn on Voltage (V)** | **Max EL Peak (nm)** | **Max Luminance (cd m^-2^)** | **Current efficiency (cd A^-1^)** | **Max EQE (%)** | **CIE x** | **CIE y** |
| --- | --- | --- | --- | --- | --- | --- | --- |
| Pristine LED | 4.75 | 512 | 166 | 0.11 | 0.31 | 0.082 | 0.736 |
| SHP LED | 2.75 | 512 | 1310 | 1.17 | 6.10 | 0.076 | 0.719 |
| ISHP LED | 2.75 | 512 | 1410 | 20.67 | 8.43 | 0.079 | 0.730 |

**Table S8.** Comparison of ISHP LED device with other recently published works.

| **S.No** | **Emission layer** | **Luminance**  **(cd m^-2^)** | **EL peak (nm)** | **Current efficiency (cd A^-1^)** | **EQE (%)** | **Operational**  **Lifetime**  **(T_50%_)** | **Bending radius** | **Ref.** |
| --- | --- | --- | --- | --- | --- | --- | --- | --- |
| 1 | CsPbBr_3_+  P123 | 3390 | 516/- | 39.2 | 11.9 | 30 mins | 4.45 mm | ^1^ |
| 2 | CH_3_NH_3_PbBr_3_/PEO | 8885 | - | 2.01 | 0.80 | - | 3.5 mm | ^2^ |
| 3 | FA_0.8_Cs_0.2_PbBr_3_ NCs | 5358 | 532/23 | 11.37 | 2.58 | - | - | ^3^ |
| 4 | CH_3_NH_3_PbBr_3_ | 2434 | - | 1.05 | - | - | - | ^4^ |
| 5 | 2D FAPbBr_3_ + PMMA | 2755 | 530/22.6 | 6.16 | 1.43 | - | 7.5mm/ 2 mm | ^5^ |
| 6 | MAPbBr_3_-QW | 31667 | 509/- | 22 | 7.3 | 18.2 hrs | - | ^6^ |
| 7 | MAPbBr_3_-QD | 3187 | 532/24 | 9.2 | - | - | - | ^7^ |
| 8 | CH_3_NH_3_PbBr_3_ QDs | 6960 | 524/24 | 10.5 | 2.7 | - | 4 mm | ^8^ |
| 9 | Q-2D BA_2_FA_2_Pb_3_Br_10_ | 1060 | 532/21 | 17.9 | 3.98 | - | 2.5mm | ^9^ |
| 10 | Q-2D CsPbBr_3_ | 25972 | 513 | 25.1 | 8.0 | - | - | ^10^ |
| 11 | Q-2D FA Cs mixed Perovskite + PETA+ PS | 32000 | 532 |  | 22.1 | 52 mins | 5mm | ^11^ |
| 12 | MAPbBr_3_ | 1260 | - | 0.79 | 0.17 | - | 1mm | ^12^ |
| 13 | K_4_MnBr_1.33_Cl_4.67_ | 2800 | 625/45 | - | 4.9 | - | - | ^13^ |
| 14 | CsPbI_3_ NCs | 306.4 | 660/37 |  | 19.8 | 8.3 mins | - | ^14^ |
| 15 | Q-2D FA perovskite | 3936 | 529/25 | 25.13 | 5.91 | - | - | ^15^ |
| 16 | Q-2D SHP | 1040 | 512/21 | 20.67 | 8.43 | 310 mins | 5mm | This work |

P123- polyethylene oxide-polypropylene oxide-polyethylene oxide triblock copolymer

PETA- pentaerythritol tetraacetate

PS- Polystyrene

**References**

1.	Sun K*, et al.* Crystallization Regulation and Defect Passivation of High-Performance Flexible Perovskite Light-Emitting Diodes Based on Novel Dielectric/Metal/Dielectric Transparent Electrodes. *Advanced Optical Materials* **12**, 2301752 (2024).

2.	Zhao J, Lo L-W, Wan H, Mao P, Yu Z, Wang C. High-Speed Fabrication of All-Inkjet-Printed Organometallic Halide Perovskite Light-Emitting Diodes on Elastic Substrates. *Adv Mater* **33**, 2102095 (2021).

3.	Zhang Q*, et al.* Highly efficient organic-inorganic hybrid perovskite quantum dot/nanocrystal light-emitting diodes using graphene electrode and modified PEDOT:PSS. *Organic Electronics* **72**, 30-38 (2019).

4.	Jung DH*, et al.* Flash-induced ultrafast recrystallization of perovskite for flexible light-emitting diodes. *Nano Energy* **61**, 236-244 (2019).

5.	Kumar S*, et al.* Ultrapure Green Light-Emitting Diodes Using Two-Dimensional Formamidinium Perovskites: Achieving Recommendation 2020 Color Coordinates. *Nano Letters* **17**, 5277-5284 (2017).

6.	Zhang D*, et al.* Large-scale planar and spherical light-emitting diodes based on arrays of perovskite quantum wires. *Nature Photonics* **16**, 284-290 (2022).

7.	Li Y-F*, et al.* Stretchable Organometal-Halide-Perovskite Quantum-Dot Light-Emitting Diodes. *Adv Mater* **31**, 1807516 (2019).

8.	Zhao F*, et al.* Highly flexible organometal halide perovskite quantum dot based light-emitting diodes on a silver nanowire–polymer composite electrode. *Journal of Materials Chemistry C* **5**, 531-538 (2017).

9.	Lee SY*, et al.* Highly Efficient Flexible Perovskite Light-Emitting Diodes Using the Modified PEDOT:PSS Hole Transport Layer and Polymer–Silver Nanowire Composite Electrode. *ACS Applied Materials & Interfaces* **11**, 39274-39282 (2019).

10.	Liu L*, et al.* Photopatternable and Highly Conductive PEDOT:PSS Electrodes for Flexible Perovskite Light-Emitting Diodes. *ACS Applied Materials & Interfaces* **15**, 21344-21353 (2023).

11.	Liu C*, et al.* Constructing Multi-Functional Polymeric-Termination Surface Enables High-Performance Flexible Perovskite LEDs. *Adv Funct Mater* **34**, 2404791 (2024).

12.	Lee SY*, et al.* Flexibility of Semitransparent Perovskite Light-Emitting Diodes Investigated by Tensile Properties of the Perovskite Layer. *Nano Letters* **19**, 971-976 (2019).

13.	Cai Y*, et al.* Suppressing the Jahn-Teller Effect in 0D Manganese-Based Perovskite Derivatives for Transparent and Flexible Light-Emitting Diodes. *Advanced Optical Materials* **13**, 2402650 (2025).

14.	Lee K*, et al.* Highly efficient pure red light-emitting diodes through surface bromination of CsPbI3 perovskite nanocrystals for skin-attachable displays. *Materials Today* **75**, 2-10 (2024).

15.	Kim H, Ra HN, Kim JS, Paek S-H, Park J, Kim YC. Improved performance of flexible perovskite light-emitting diodes with modified PEDOT:PSS hole transport layer. *Journal of Industrial and Engineering Chemistry* **90**, 117-121 (2020).
